# MiR-224 Targets the 3′UTR of Type 1 5′-Iodothyronine Deiodinase Possibly Contributing to Tissue Hypothyroidism in Renal Cancer

**DOI:** 10.1371/journal.pone.0024541

**Published:** 2011-09-02

**Authors:** Joanna Boguslawska, Anna Wojcicka, Agnieszka Piekielko-Witkowska, Adam Master, Alicja Nauman

**Affiliations:** Department of Biochemistry and Molecular Biology, The Medical Centre of Postgraduate Education, Warsaw, Poland; Cardiff University, United Kingdom

## Abstract

Type 1 iodothyronine deiodinase (DIO1) catalyses the conversion of prohormone thyroxine to the active thyroid hormone 3,3′,5-triiodothyronine (T3), important regulator of cell proliferation and differentiation. DIO1 expression is reduced in the most common type of kidney neoplasia, clear cell Renal Cell Carcinoma (ccRCC). MicroRNAs are small, non-coding RNAs that regulate gene expression at posttranscriptional levels. The aim of this study was to analyze the potential regulation of DIO1 expression by microRNAs in ccRCC. Bioinformatic analysis revealed that 3′UTR of the human *DIO1* gene transcript contains miR-224 and miR-383 target sites, which are conserved across mammalian species. Semi-quantitative real-time PCR was used to analyze the expression of miR-224 and miR-383 in 32 samples of ccRCC tumors (T) and in 32 matched control (C) samples. We observed statistically significant (p = 0.0002) more than four fold increase in miR-224 expression and nearly two fold increase in miR-383 expression in samples T compared to samples C. Tumor specific changes in expression of miR-224 negatively correlated with changes in DIO1 expression and intracellular T3 concentration. Transfection of HeLa cell line with miR-224 and miR-383 suppressed the activity of a luciferase reporter containing the 3′UTR of *DIO1*. This was abolished when constructs mutated at the miR-224 and miR-383 target sites were used instead, indicating that miR-224 and miR-383 directly bind to *DIO1* 3′UTR. Finally, induced expression of miR-224 in Caki-2 cells resulted in significant (p<0.01) reduction of *DIO1* mRNA. This study provides a novel miRNA-mediated regulatory mechanism of *DIO1* expression in ccRCC.

## Introduction

Thyroid hormones: 3,5,3′-L-triiodothyronine (T3) and thyroxine (T4), play important roles in growth, development, differentiation, and regulation of metabolic pathways in cells. Human type 1 iodothyronine deiodinase (DIO1), product of the *DIO1* gene catalyzes two types of deiodination reaction, an outer-ring (5′-deiodination - 5′D) and an inner-ring (5-deiodination - 5D). These processes result, respectively, in the activation and inactivation of thyroid hormones (1). DIO1 is a selenoenzyme expressed mainly in liver, kidney, thyroid, and pituitary. Previous reports have shown that expression of this enzyme is disturbed in different types of cancer. For instance, *DIO1* mRNA and activity are decreased in papillary thyroid carcinoma (2–5) and increased in follicular adenoma and follicular thyroid carcinoma (2). In previous works we showed decreased expression of *DIO1* mRNA and activity (6), and disturbed alternative splicing of *DIO1* pre-mRNA in clear cell Renal Cell Carcinoma (ccRCC) (7). *DIO1* expression has also been proposed as a differentiation marker of cancer cells (8, 9).

ccRCC represents the most common renal cancer histology, representing 75% of primary malignancies of the kidney (10, 11). The commonly used treatment is surgical resection while chemo- and radiotherapy remain inefficient. None of the several proposed molecular markers has been approved for clinical use (10).

MicroRNAs (miRNAs) are small, non-coding RNAs that control genes expression by completely or partially complementarily binding to the 3′-untranslated region of target mRNA (12, 13) causing degradation of the mRNA or, more commonly, blocking translation (14–16). Previous studies have shown that miRNAs play important roles in essential processes, such as differentiation, proliferation, and apoptosis (17, 18). Currently emerging results revealed that miRNAs are involved in cancer pathogenesis. Frequent alterations of miRNA expression have been found in a variety of human malignancies such as thyroid (19), lung (20), pancreas (21), colon (22), breast (23), liver (24), prostate (25) or other solid tumors (26, 27). Various studies have identified panels of microRNAs that are differentially expressed between normal renal tissue and tumor or between histological subtypes of renal tumor (28–33). MicroRNA expression profiling has shown diverse clinical applications for diagnosis, prognosis and predictive purposes (34, 35). Several miRNAs function as oncogenes or tumor suppressors, and multiple genes coding for miRNAs are located in genomic regions involved in cancers (36).

Our previous results (6) have shown that DIO1 activity poorly correlates with its mRNA level in healthy renal tissues. This observation suggests significant posttranscriptional regulation of *DIO1* expression that could be mediated by miRNAs. Therefore the aim of this work was to investigate the potential *DIO1* regulation by miRNAs and to determine whether the deregulation of DIO1 in ccRCC could result from altered actions of miRNAs.

## Results

### Computational prediction of miRNAs binding to *DIO1* 3′UTR

To identify the putative miRNA targeting of the 3′UTR of *DIO1* mRNA, we used computational programs, TargetScan, PicTar, miRBase and miRANDA. The 1087 nucleotides of 3′UTR of *DIO1* were screened for complementarity to seed sequences of known miRNAs. Only the miRNAs identified by at least two of four independent bioinformatics approaches were considered for further analysis. We identified 7 potential miRNAs targeting the 3′UTR of human *DIO1* (Table 1): miR-224, miR-383, miR-610, miR-659, miR-637, miR-1202 and miR-1266.

**Table 1:** Potential binding sites for microRNAs in 3′UTR of *DIO1* as determined by computational tools: TargetScan (T); miRBase (B); PicTar (P); miRanda (R).

miRNA	Coordinates	Mature sequence	Prediction method
hsa-miR-224	X:151127050-151127130[−]	CAAGUCACUAGUGGUUCCGUU	T, P, R
hsa-miR-383	8:14710947-14711019 [-]	AGAUCAGAAGGUGAUUGUGGCU	T, P, B
hsa-miR-610	11:28078362-28078457[+]	UGAGCUAAAUGUGUGCUGGGA	T, R
hsa-miR-637	19:3961412-3961510[−]	ACUGGGGGCUUUCGGGCUCUGCGU	B, R
hsa-miR-659	22:38243685-38243781[−]	CUUGGUUCAGGGAGGGUCCCCA	T, B
hsa-miR-1202	6:156267931-156268013[+]	GUGCCAGCUGCAGUGGGGGAG	T, R
hsa-miR-1266	15:52569314-52569397[+]	CCUCAGGGCUGUAGAACAGGGCU	T, R

### miR-224 and miR-383 are overexpressed in ccRCC

In preliminary studies, using semi quantitative real-time PCR (SQ-PCR), we determined the relative expression of 7 candidate miRNAs in ccRCC and paired match control samples from 32 patients using universal primer UniAmpHindIII (37) with sequence homology to overhangs of primers used in reverse transcription and miRNA-specific primers (sequences are shown in Supplemental data, in Table S2). We observed differing expression of miR-224 and miR-383, whereas expression of the five other candidate miRNAs: miR-610, miR-637, miR-659, miR-1202 and miR-1266 did not differ significantly between ccRCC and control tissue (Figure S1, Supplemental Data).

**Figure 1:**
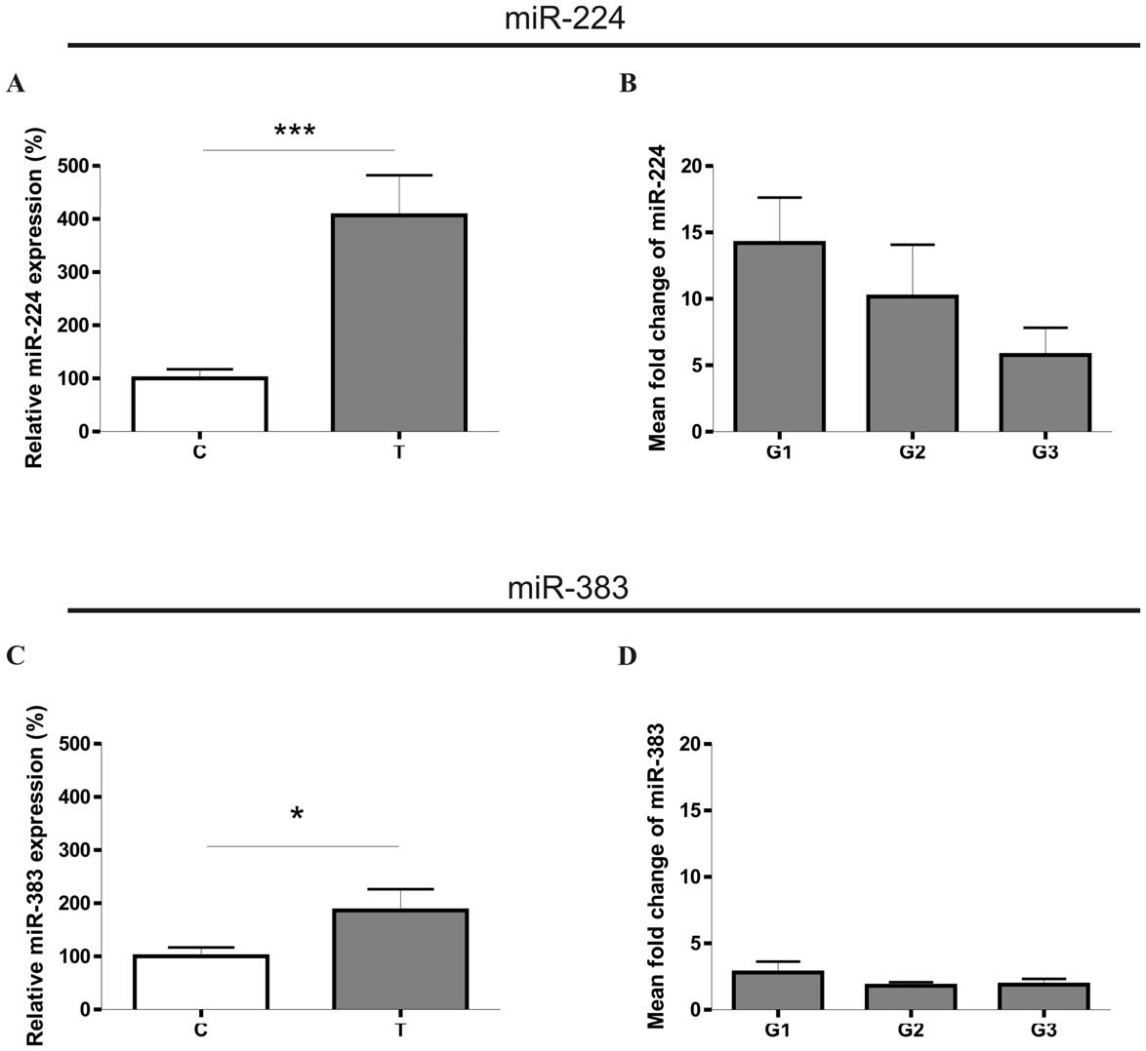
Expression of miR-224 (A and B) and miR-383 (C and D) in ccRCC. **A.** Increased miR-224 expression in ccRCC tumor samples (T) compared with control samples (C). The expression is shown as percentage of control C. **B.** Mean fold T: C change of expression of miR-224 224 in samples divided according to tumor differentation grades (G1, G2, G3). **C.** Increased miR-383 expression in ccRCC tumor samples (T) compared to control samples (C). The expression is shown as percentage of control C. **D.** Mean fold T: C change of expression of miR-383 224 in samples divided according to tumor differentation grades (G1, G2, G3). Data are given as mean ± SEM n = 32 for T, n = 32 for C (in A and C), n = 11 for G1, n = 11 for G2, n = 10 for G3 (in B and D). Statistical analysis was performed using paired *t*-test to compare C and T samples (in A and C) or ANOVA to compare G1, G2, and G3 samples (in B and D)* p<0.05, *** p<0.001.

Induced expression of miR-224 and miR-383 in ccRCC was confirmed in a specific TaqMan microRNA assay. These studies revealed statistically significant (p = 0.0002) over four fold increase in the expression of miR-224 and nearly two fold increase (p = 0.0236) in the expression of miR-383, in samples T compared to control samples C (Figure 1). Mean fold change of miR-224 and miR-383 was analyzed in different tumor gradings but did not depend on the differentiation grade of tumor sample. However, mean fold change of miR-224 tended to decrease when differentiation grades increased from G1 to G3 (Figure 1).

Thus, disturbed expression of miR-224 and miR-383 in ccRCC was confirmed by two different analytical approaches.

### miR-224 negatively correlates with DIO1 and T3 levels in ccRCC

**Figure 2:**
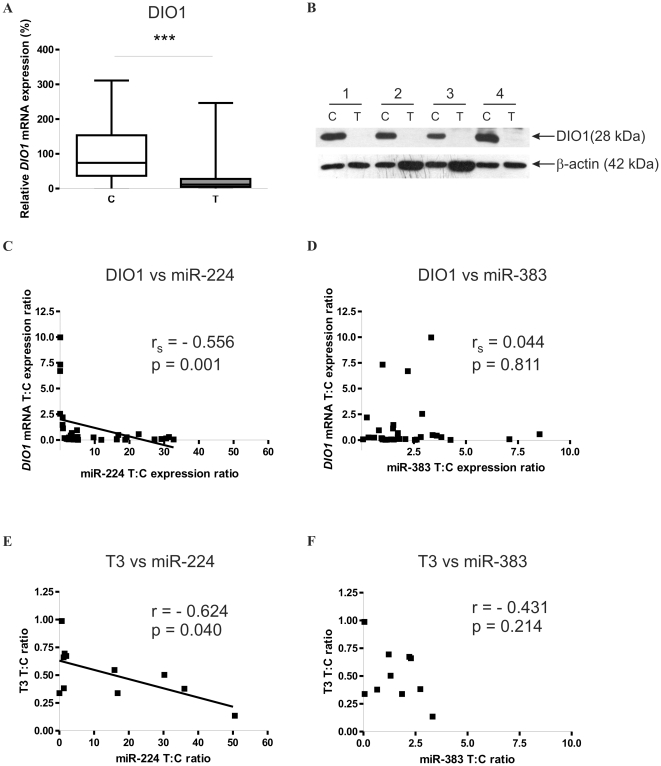
miR-224 expression negatively correlates with *DIO1* mRNA and T3 in ccRCC. **A.** Expression of *DIO1* mRNA in tissue samples. n = 32 for T and n = 32 for C. The expression is shown as percentage of control C. The plot shows median values with 95% CI as data were not normally distributed. Statistical analysis was performed using Wilcoxon paired test to compare C and T samples. *** p<0.001. B. Western-blot of DIO1 performed on pair-matched control-ccRCC samples. β-actin expression was used as an internal control. Four representative control (C) and tumor (T) samples are shown. **C and D.** Scatter plots of the T: C ratios of *DIO1* mRNA *versus* T: C miR-224 (C) and T: C miR-383 (D) expression ratios. Non-parametric Spearman's rank correlation analysis was performed on data from 32 pairs of control and tumor tissue samples. p<0.05 was considered statistically significant. **E and F.** Scatter plots of T3 concentration T: C ratio versus T: C miR-224 (E) and miR-383 (F) expression ratios. Pearson correlation analysis was performed on data from 11 pairs of control and tumor tissue samples. p<0.05 was considered statistically significant.

SQ-PCR analysis revealed 2.7 fold downregulation of *DIO1* mRNA in 32 paired tumor and control tissue samples (p = 0.0009), which is consistent with our previous reports (6) (Figure 2). As shown in Figure 2, statistically significant negative correlation was observed between miR-224 and *DIO1* mRNA tumor-specific changes of expression (Spearman r_s_ = −0.556 at p = 0.001). In contrast, no correlation was observed for miR-383 and *DIO1*. Moreover, Western-blot analysis performed on eleven paired samples of ccRCC and normal kidney tissue revealed loss of DIO1 protein (Figure 2B).

DIO1 is a regulator of thyroid hormone bioavailability. In our recent study we found that T3 concentration was decreased in ccRCC tumors compared with pair matched controls. To check whether miR-224-mediated downregulation of DIO1 affects DIO1 activity product, T3, we analyzed the correlation between tumor-specific changes of miR-224 and T3 levels. T3 levels were analyzed in 11 pair-matched ccRCC and control samples that were used for DIO1 Western-blot analysis. T3 concentration was assessed as described previously (37). We found a statistically significant (Pearson r = −0.624, p = 0.04) negative correlation between T: C ratios of miR-224 and intratumoral T3 (Fig. 2). miR-383 changes did nor correlate with T3.

Lowered intratumoral T3 concentrations may result not only from decreased expression of DIO1 but also from increased activity of type 3 deiodinase (DIO3) which is a thyroid hormone inactivating enzyme. Using Q-PCR, we analyzed expression of DIO3 mRNA in 11 pair-matched ccRCC and control samples. DIO3 mRNA was undetectable in all the analyzed samples (results not shown). Thus, we confirmed that decreased intratumoral T3 concentrations result from lowered DIO1 expression.

### Transfection with miR-224 decreases expression of endogenous *DIO1*

**Figure 3:**
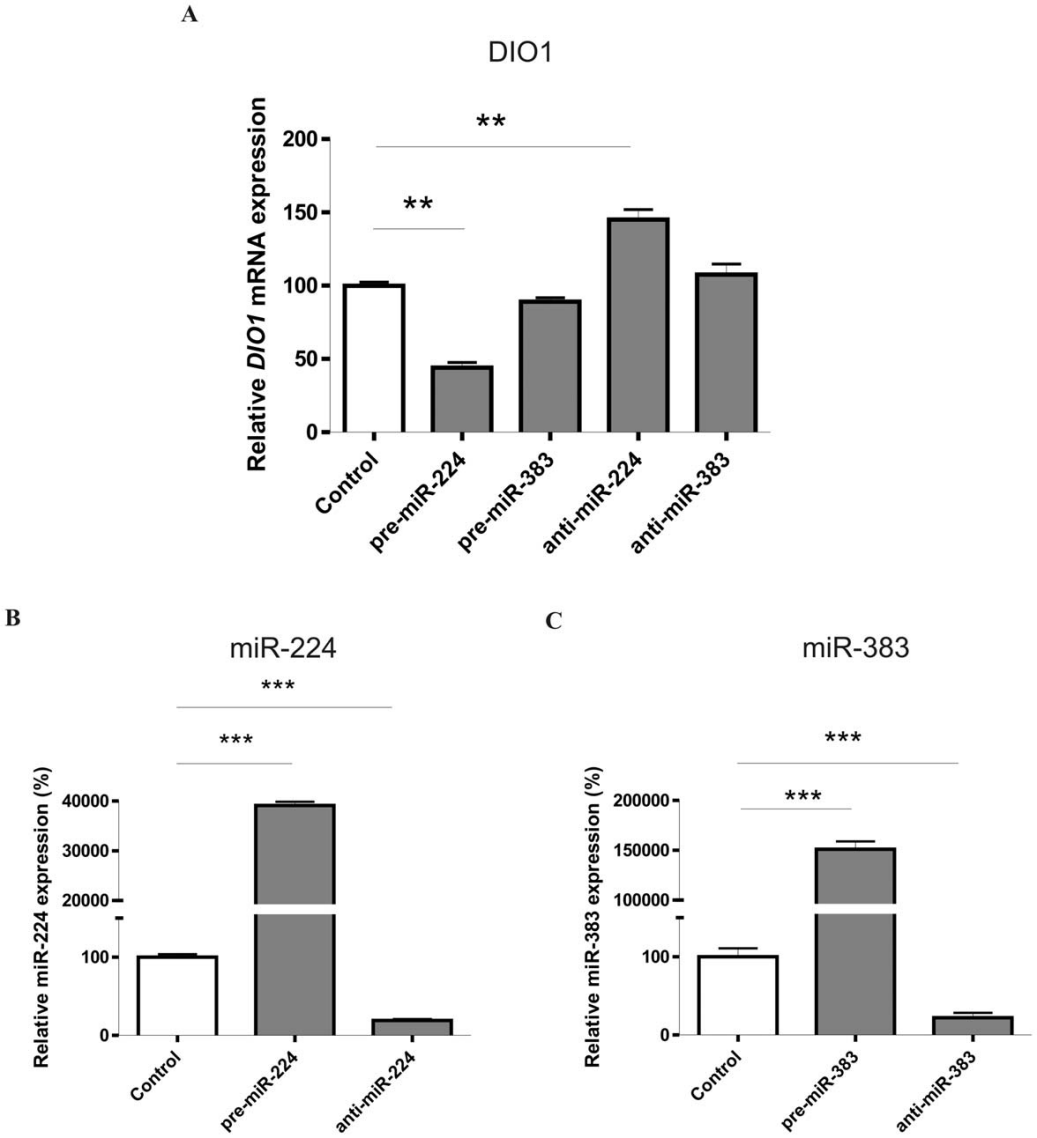
miR-224 regulates endogenous *DIO1* expression in Caki-2 cell line. **A.** Expression of *DIO1* mRNA in the Caki-2 cell line. Cells were transfected with 37.5 pmoles of pre-miRs, anti-miRs or scrambled pre-miR (negative control); after 48 h total RNA was extracted for subsequent SQ-PCR analysis of *DIO1* level. The expression is shown as percentage of control (cells transfected with scrambled microRNA). Data are given as mean ± SEM. Data were analyzed by ANOVA followed by Dunnett's multiple comparison test. ** p<0.01. **B and C.** The level of miR-224 (B) and miR-383 (C) in cells transfected with pre-miRs or anti-miRs. Data are presented as percentage of control (cells transfected with scrambled microRNA). Data are given as mean ± SEM Values for miRNAs were normalized to U6 gene. Data were analyzed by ANOVA followed by Dunnett's multiple comparison test. *** p<0.001.

To determine the functional effect of miR-224 and miR-383 on endogenous *DIO1* mRNA, Caki-2 cells were transfected with pre-miR-224, pre-miR-383 (microRNA precursors), anti-miR-224, anti-miR-383 (microRNA inhibitors), or scrambled control. Successful transfection was confirmed in SQ PCR analysis by a large induction of miR-224 and miR-383 after transfection with microRNA precursors and significant decrease of miRNAs expression when inhibitors were used (Figure 3).

mRNA levels of *DIO1* were measured by SQ-PCR and a significant reduction (56%, p<0.01) of the *DIO1* transcript level was observed after introduction of pre-miR-224. pre-miR-383 did not have this effect (Figure 3). Transfection of Caki-2 cells with anti-miR-224 resulted in increase of *DIO1* expression, by 45%, p<0.01, when compared with scrambled control. We did not observe this overexpression when cells were transfected with anti-miR-383 (Figure 3).

These data show that endogenous *DIO1* mRNA expression in Caki-2 cells is modulated by miR-224.

### The 3′UTR of *DIO1* is a direct target for miR-224 and miR-383

**Figure 4:**
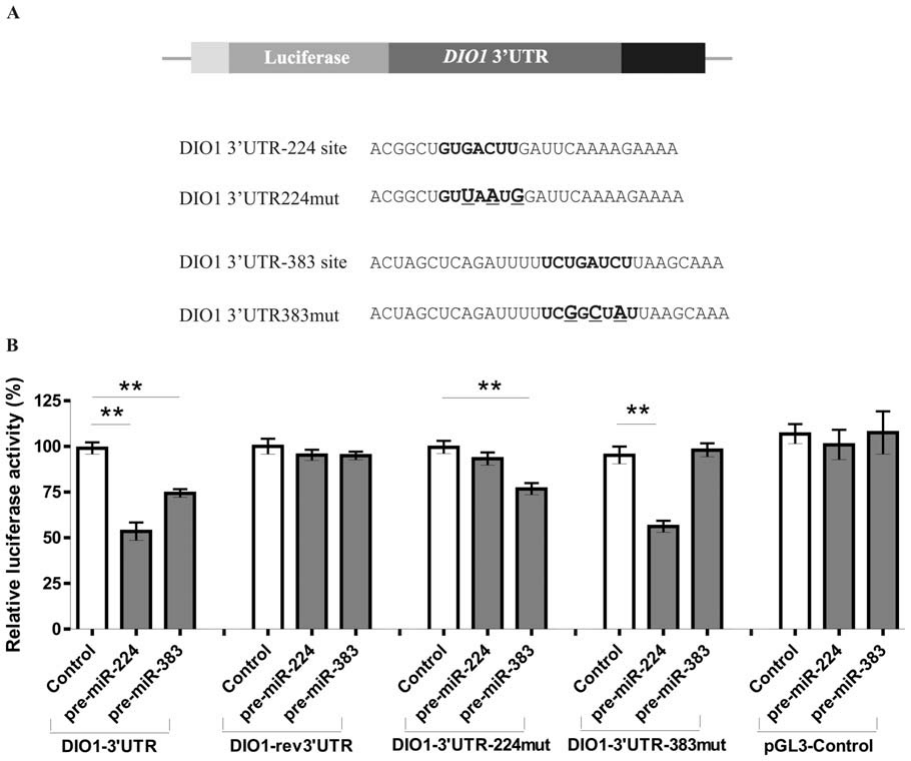
The *DIO1* 3′UTR is the direct target for miR-224 and miR-383. **A.** *DIO1* 3′UTR cloned downstream of luciferase in the pGL3-Control vector. The wild-type and mutated 3′UTR of *DIO1* with the seed region (bold) and base substitutions (large font and underlined) abolishing binding of a particular miRNA (verified *in silico*) are presented below. Two types of 3′UTR mutants were constructed: *DIO1*-3′UTR224mut and *DIO1*-3′UTR383mut. **B.** Dual luciferase assay. The relative luciferase activity of constructs with the 3′UTR of *DIO1*: *DIO1*-3′UTR, *DIO1*-rev3′UTR, *DIO1*-3′UTR224mut, *DIO1*-3′UTR383mut and pGL3-Control in the presence of pre-miRNA or scrambled pre-miRNA (negative control). Data are expressed as mean values ± SEM and are shown as percentage of control (cells transfected with scrambled microRNA). Each bar represents values from three independent experiments, measured in triplicates. The relative activity of Firefly luciferase expression was normalized to Renilla luciferase activity. Data were analyzed by ANOVA followed by Dunnett's multiple comparison test. ** p<0.01.

Computational analysis of *DIO1* 3′UTR with TargetScan5.1 revealed two putative binding sites for miR-224 and miR-383, located at nucleotides 1788–1794 and 898–904 of *DIO1* transcript (NM_00792.5), respectively (Figure S2 in Supplemental Data). These two sites were conserved across mammalian species. To study the direct interaction between the miR-224, miR-383 and *DIO1* transcript we cloned the 3′UTR of *DIO1* downstream of the luciferase reporter gene in the pGL3-control vector – (DIO1-3′UTR). Control plasmid, in which DIO1 3′ UTR was inserted in a reverse orientation (DIO1-rev3′UTR) was also constructed. In parallel, we created two additional reporter constructs in which the conserved targeting regions were specifically mutated, to abolish binding of miRNAs (Figure 4). HeLa cell line, exhibiting low endogenous expression of *DIO1* was used for transfection experiments. Cells were cotransfected with obtained constructs and precursors of microRNAs: pre-miR-224, pre-miR-383 or control (scrambled miRNA). In HeLa cells transiently transfected with the *DIO1*-3′UTR construct and miRNA precursors, a significant inhibition of luciferase activity was observed. Both miR-224 and miR-383 caused decrease in luciferase activity by about 45% and 25%, respectively, compared with the control scrambled miRNA. Both pre-miRNAs failed to exert a significant effect on *DIO1*-rev3′UTR and the empty pGL3-Control vector (Figure 4).

The luciferase activity of the reporter constructs: *DIO1*-3′UTR224mut and *DIO1*-3′UTR383mut, in which target sites recognised by the microRNA were mutated, was unaffected by transfection with pre-miR-224 or pre-miR-383, respectively (Figure 4). This observation confirmed the specificity of action of both microRNAs as mutation in the recognition site should prevent from miRNA binding to 3′UTR. What is more important, mutation in the recognition site of one microRNA did not influence binding of the other analyzed miRNA. The luciferase activity in cells transfected with *DIO1*-3′UTR383mut was inhibited by 40% after pre-miR-224 addition, whereas transfection with *DIO1*-3′UTR224mut and pre-miR-383 resulted in ∼23% decrease in luciferase activity (Figure 4).

Taken together, these data show that the 3′UTR of *DIO1* contains specific binding sites for microRNAs miR-224 and miR-383.

## Discussion

In this study we have shown for the first time that type 1 iodothyronine deiodinase transcript is a direct functional target for microRNA miR-224. Binding of miR-224 to *DIO1* 3′UTR results in downregulation of endogenous *DIO1* expression in renal cancer cells. Moreover, *DIO1* 3′UTR contains specific conserved binding sites for miR-224 and miR-383, as showed in luciferase reporter assays. Both microRNAs are markedly overexpressed in clear cell renal cancer and possibly account for loss of *DIO1* expression in tumor. This has been supported by the negative correlation between tumor-specific changes in miR-224 and *DIO1* mRNA levels and loss of DIO1 protein in ccRCC samples. Moreover, tumor-specific changes in concentration of product of DIO1 activity, T3, correlate negatively with changes of miR-224 expression. These results provide a strong evidence for a novel mechanism of *DIO1* regulation.

The mechanisms regulating *DIO1* expression are poorly understood. In our previous studies we observed lack of correlation between DIO1 protein and mRNA level in ccRCC (6) which suggested the possible involvement of posttranscriptional mechanisms, such as microRNA-dependent regulation. In the present study we observed nearly 3-fold reduction of *DIO1* mRNA expression while DIO1 protein was lost in tumor samples. An interesting observation made in the present study is that the expression of miR-224 tends to change with tumor differentiation grades and is highest in G1 and lowest in G3 (Fig. 2). Although these changes between the respective tumor differentiation grades are not statistically significant they may possibly suggest that as tumor progress the impact of miR-224 on *DIO1* expression lowers and perhaps other posttranscriptional mechanisms are involved. As we have previously shown, another mechanism contributing to impaired DIO1 expression in ccRCC is its disturbed alternative splicing resulting possibly from changes in expression of splicing factors (7, 38). In the experiments performed in Caki-2 cells, miR-383 did not affect the expression of endogenous DIO1. This may suggest that miR-383 acts on a posttranscriptional level, causing translation blockage rather than degradation of *DIO1* mRNA. The second possibility is that the other factors (including miR-224) may exert stronger effect on DIO1 expression in ccRCC. This idea is supported by results of luciferease experiments in which induced miR-383 expression resulted in only 25% reduction of luciferase activity in comparison with the effect of miR-224 which caused 45% downregulation of expression. Luciferase experiments confirm the hypothesis that binding of miR-383 to *DIO1* 3′UTR results in translation blockage, as luciferase activity is a direct consequence of actual luciferase protein levels.

Since altered expression of miR-224 and miR-383 was observed in tumors of tissues expressing type 1 iodothyronine deiodinase it suggests that these tumors may also present disturbed expression of *DIO1*. Indeed, in papillary thyroid cancers, expression of miR-224 was significantly elevated (39) while our studies revealed that in this cancer, expression of *DIO1* is reduced (3). Conversely, in breast cancer, miR-383 is lost (40) while *DIO1* expression significantly increases (41). Whether impaired expression of *DIO1* truly results from altered miR-224 and miR-383 levels in these cancers needs to be evaluated by separate studies.

The significance of our findings comes from the role of DIO1 in cellular physiology. DIO1 is an enzyme regulating bioavailability of thyroid hormones. Recently it was suggested that DIO1 does not contribute to circulating T3 levels but rather acts as a scavenger enzyme involved in iodide recycling (42). However, the possibility that DIO1 contributes to locally synthesized T3 in DIO1 expressing tissues can not be ruled out as it was previously suggested (43, 37). Thus, microRNAs regulating DIO1 expression in ccRCC may have the potential influence on intratumoral thyroid hormone levels. Indeed, as we demonstrate in the present study, the tumor-specific changes in intracellular T3 concentration correlate with changes of miR-224 expression. Interestingly, in our recent study we found that transcript of thyroid hormone receptor beta gene (*THRB*) is also a target for microRNA-dependent regulation (37). miR-204 is overexpressed in ccRCC and results in concomitant downregulation of *THRB* expression. These results together with findings of the present study suggest that microRNAs may possibly contribute to tissue hypothyroidism in ccRCC resulting in downregulation of key genes of thyroid hormone pathway, *THRB* and *DIO1* and, in consequence, leading to the the decrease of intratumoral T3 levels. This is an important observation since several studies demonstrated that THRB can act as a tumor suppressor and that hypothyroidism may influence tumor growth (44–48). Thus, microRNA-dependent regulation of intracellular thyroid state may possibly have the potential to affect neoplastic process. Interestingly, this may be a more general phenomenon in tumors with disturbed expression of *DIO1* and *THRB*. In our recent study several microRNAs that were upregulated in papillary thyroid tumors (PTC) were shown to directly target THRB transcript resulting in significant downregulation of its expression (49). It would be of interest to check whether the expression of microRNAs targeting *DIO1* is also disturbed in PTC tumors. Our previous studies showed that loss of DIO1 expression in PTC is accompanied by lack of correlation between mRNA and protein expression (3). This suggests possible posttranscriptional regulation including microRNA involvement. However, whether impaired *DIO1* expression in PTC results from microRNA-mediated deregulation needs to be verified by further studies.

Disturbed microRNA expression in ccRCC has already been reported in other studies. Interestingly, among microRNAs differently expressed in control and tumor samples miR-224 has been repeatedly found by independent studies (30, 33, 50). This suggests the possible use of miR-224 as a marker differentiating ccRCC tumors from healthy tissues. Regarding miR-383, it was reported as downregulated in hepatocellular carcinoma (51), breast and ovarian cancers, melanoma (40), acute myeloid leukemia (52) and central nervous system tumors (53). Thus, our work is the first one showing upregulation of miR-383 in cancer. Whether this is a specific feature of kidney neoplasia remains to be verified by future experiments. Both microRNAs, miR-224 and miR-383 are believed to be implicated in control of proliferation or apoptosis. miR-224 was shown to increase apoptotic cell death and proliferation in hepatocellular carcinoma (54) while overexpression of miR-383 inhibited proliferation of testicular embryonic carcinoma cells (55). The question whether miR-224 and miR-383 are involved in ccRCC proliferation awaits future studies.

In summary, we demonstrated that *DIO1* 3′UTR is targeted by two microRNAs: miR-224 and miR-383. miR-224 mediates loss of DIO1 in renal cancer, what results in decreased intratumoral T3 concentration. These results provide a strong evidence for a new mechanism regulating the expression of type 1 iodothyronine deiodinase. Previous reports revealed that disturbed expression of THRB, another gene of thyroid hormone pathway, observed in ccRCC, may also result from microRNA-dependent deregulation. Together, these results suggest that the new class of small, non-coding RNAs may possibly contribute to intracellular hypothyroidism in ccRCC.

## Materials and Methods

### Tissue samples and cell lines

Tissue samples were obtained with the permission of the Bioethics Committee of the Medical Centre of Postgraduate Education in Warsaw from patients with clear cell renal cell carcinoma (32 patients). Written informed consent was obtained from all patients involved in this study. Samples were divided into two groups: tumor samples (n = 32, T) and control samples (paired normal tissue from the opposite pole of the malignant kidney with no histological evidence of tumor; n = 32, C). Clear cell renal cell carcinoma was diagnosed histologically according to WHO criteria (56). Tumors were divided into three groups depending on the grade of differentiation: G1 (well differentiated), G2 (intermediate grade of differentiation), G3 (poorly differentiated cancers).

Cervical cancer (HeLa) and clear cell renal cell cancer (Caki-2) cell lines used in this study were purchased from the American Type Culture Collection, (USA) and cultured according to the ATCC protocol. The cells were seeded into 12 well culture plates at density 5×10^4^ (Caki-2) or 1×10^5^ (HeLa) cells/well 24 h before transfection.

### Luciferase Reporter Constructs

1023 bp fragment of *DIO1* was amplified using cDNA from HeLa cell line (primers *DIO1*-3′UTR F and R, Table S1, Supplemental data), cloned into XbaI-site immediately downstream of the stop codon in the pGL3-Control Firefly Luciferase reporter vector (Promega, USA), sequenced and named *DIO1*-3′UTR, or *DIO1*-rev3′UTR, depending on the orientation of the cloned insert. The reversely inserted *DIO1*-rev3′UTR was used as negative control vector. Site-directed mutagenesis of the miR-224 and miR-383 target sites: GTGACTT of miR-224 within nt 1788–1794 and TCTGATCT of miR-383 within nt 898–904 in the *DIO1* 3′UTR was performed using Quick change-mutagenesis kit (Stratagene, Germany). Primers Mut224 F/R and Mut383 F/R (Table S1, Supplemental data) and *DIO1*-3′UTR plasmid as a template were used. Two constructs: *DIO1*-3′UTR224mut and *DIO1*-3′UTR383mut were obtained.

Sequencing reaction was performed using BigDye Terminator v3.1 Cycle Sequencing Kit (Applied Biosystems, USA).

### Cells transfection and luciferase assay

Caki-2 cells were seeded at 0.5×10^5^ cells per 12-well dish and transfected 24 hours later using Lipofectamine 2000 reagent (Invitrogen, USA) as described by the manufacturer with 37.5 pmoles of miRNA precursors: pre-miR-224 and pre-miR-383 (Pre-miR^™^ miRNA Precursor Molecule, Ambion, USA), miRNAs inhibitors: anti-miR-224 and anti-miR-383 (Anti-miR™ miRNA Inhibitor Molecule, Ambion, USA) or control scrambled microRNA (Negative microRNA Control, Ambion, USA). Cells were harvested after 48 h for RNA extraction.

miRNA precursors are synthetic RNA duplexes that mimic endogenous miRNAs, whereas inhibitors have a sequence complementary to mature miRNAs and function by sequestering/degrading endogenous miRNAs.

For reporter gene assays, HeLa cells were seeded at 1×10^5^ in 12-well plates and 24 h later, cotransfected with Lipofectamine 2000 reagent (Invitrogen, USA). Each cotransfection reaction contained 100 ng pRL-TK vector (Promega, USA) expressing Renilla luciferase, 1 ug of pGL-3′ UTR vectors and 37.5 pmoles of pre-miRs or scrambled microRNA. After 48 hours, cells were lysed and luciferase activity was analyzed in dual-luciferase assay (Promega, USA) using a Synergy2 luminometer (BioTek, USA). Firefly luciferase activity was normalized to Renilla luciferase activity. In all the experiments, transfection and luciferase assays were performed in triplicates.

### RNA isolation and reverse transcription

Total cellular RNA was isolated as described previously (37). Reverse transcription was performed using RevertAidTM H Minus First Strand cDNA Synthesis Kit (Fermentas, Lithuania). For reverse transcription, 200 ng of total RNA was used with Random Hexamer primers or specific stem-loop primers with 5′-overhangs (Table S2, Supplemental data) for microRNAs.

cDNA for miR-224 and miR-383 was also synthesized with specific miRNA primers from the TaqMan MicroRNA Assays (Applied Biosystems, USA) and reagents from the TaqMan MicroRNA Reverse Transcription kit (Applied Biosystems, USA).

### SQ-PCR

*DIO1* expression analysis in Caki-2 cells was performed using DNA SYBR Green I Master (Roche Diagnostics, Germany) in triplicates according to manufacturers' protocols. SQ-PCR reaction was carried out under the following conditions: 95°C for 10 min., 45 cycles: 95°C, 15 s; 57°C, 15 s; 72°C, 15 s, 68°C, 15 s; followed by melting curve analysis: 95°C, 5 min.; 65°C, 1 min.; continuous reading of fluorescence from 65°C to 97°C with 0.11°C/s ramp rate and 5 acquisitions per each °C. Results were normalized to the expression of 18sRNA host-gene *RN18S1*. *DIO1* and *DIO3* expression analysis in tissue samples was performed as described previously (37). The sequences of the primers are shown in Table S1, (Supplemental data). For *DIO3* expression, standard curve was performed using PCR product cleaned with Clean-up kit (A&A Biotechnology, Poland).

SQ-PCR quantification of miRNAs was performed using QuantiFast SYBR Green PCR Kit (Qiagen, Germany), universal primer UniAmpHindIII with sequence homology to overhangs of primers used in reverse transcription and miRNA-specific primers (sequences are shown in Supplemental data, in Table S2). Conditions for QuantiFast were as described (37).

To evaluate the expression levels of mature miRNA: 224 and 383 TaqMan MicroRNA Assay kits (Applied Biosystems, USA) were used according to the manufacturer's protocol. The assays target only mature microRNAs, not their precursors, ensuring biologically relevant results.

For normalization of miRNA expression, U6 snRNA was used as an internal control. Relative quantification of each expressed miRNA was calculated using the 2^−ΔCt^ method (57).

### Western blot analysis

Isolation of protein and Western blot analysis was described previously (37).

### Tissue T3 concentration

Intracellular T3 concentration was analyzed previously (37).

### Computational analysis

To predict miRNAs potentially binding to *DIO1* 3′UTR sequences we used commonly cited miRNA prediction programs: TargetScan5.1 (http://www.targetscan.org/) (58), miRBase (http://microrna.sanger.ac.uk/targets) (59), PicTar (http://pictar.mdc-berlin.de/) (60) and miRanda (http://microrna.org) (61). We considered microRNA as potentially binding to *DIO1* 3′UTR only if it was predicted by at least two of the four methods.

For examination of abolished binding between miRNAs and *DIO1*-3′UTR383mut or *DIO1*-3′UTR224mut a miRNA target detection tool (62) was used.

The analysis was performed based on the following reference sequences: NCBI DNA Ref. Seq.: NC_000001.10: 21969 bp (region from base 54357325 to 54379293). mRNA sequence: NM_000792.5.

### Statistical analysis

The Shapiro-Wilk test was used to determine normality of data distribution. Most data were analyzed by t-test, while *DIO1* expression in match-paired tissue samples was analyzed by Wilcoxon matched pairs test. Correlation analysis was performed with non-parametric Spearman's rank correlation test. Data from luciferase assays and transfection experiments were analyzed by ANOVA followed by Dunnett's multiple comparison test. *p<*0.05 was considered statistically significant.

## Supporting Information

Table S1 **Primers used for cloning, mutagenesis and SQ-PCR analysis.** ***SpeI*** **restriction sites in primer overhangs (underlined) are in bold.**
(DOC)

Table S2 **Primers used in analysis of miRNA. RT: primers used in reverse transcription.** PCR: primers used in SQ-PCR. F: forward, R: reverse. Uni-amp sequences are bolded.
(DOC)

Figure S1 **Expression of microRNAs predicted to bind to** ***DIO1*** **3′UTR in ccRCC.** Expression of miR-224, miR-383, miR-610, miR-637, miR-659, miR-1202 and miR-1266 was analyzed in 32 matched pairs of tumor (T) and control (C) samples. SQ-PCR reactions were performed in triplicates. The expression is shown as percentage of control C. Data are given as mean ± SEM (n = 32 for T, n = 32 for C). Statistical analysis was performed using *t*-test to compare C and T samples.
(TIF)

Figure S2 **The microRNA target sites in** ***DIO1*** **3′UTR. A**). The structure of the human *DIO1* transcript (GeneBank Acc. No. NM_00792.5). The four exons are boxed, numbers above indicate exon length. Stop codon indicates the beginning of 3′UTR. Binding sites of miR-383 and miR-224 are indicated and their positions (miR-383: nt 898-904, miR-224: nt 1788–1794) are given according to *DIO1* mRNA sequence (NM_00792.5). **B**). Bioinformatic prediction of miRNA binding sites in DIO1 3′UTR, conserved among six mammalian species performed with TargetScan5.1.
(TIF)
