# Supplementary material for: MiR-224 Targets the 3′UTR of Type 1 5′-Iodothyronine Deiodinase Possibly Contributing to Tissue Hypothyroidism in Renal Cancer
Source: PLoS One. 2011 Sep 2;6(9):e24541. doi: 10.1371/journal.pone.0024541 (PMC3166326; doi:10.1371/journal.pone.0024541)
Supplement: Table S1 — Primers used for cloning, mutagenesis and SQ-PCR analysis. SpeI restriction sites in primer overhangs (underlined) are in bold. (DOC) [file pone.0024541.s001.doc]

**Table S1.**

| Target Gene | PRIMER NAME | Primer SEQUENCE |
| --- | --- | --- |
| *DIO1* | DIO1-F | GAGGAATGCAGGCCACAGC |
|  | DIO1-R | AGAGAGATACCGTACCTCAG |
| *DIO1*-3’UTR | DIO1-3’UTR-F | ACGCA**ACTAGT**TCTGGACAGATACCTCAATTC |
|  | DIO1-3’UTR-R | ACGCA**ACTAGT**TCAAGTCACAGCCGTGTGTAC |
| *DIO3* | DIO3-F | AACGGACAATTGACTGAACTTGG |
|  | DIO3-R | GATTGTTCATGTGGCTGAGG |
| Mut224 | Mut224-F | CACAGCCTAAAGTACACACGGCTGTTAATGGATTCAAAGAAAATGTTATAAGATG |
|  | Mut224-R | CATCTTATAACATTTTCTTTTGAATCCATTAACAGCCGTGTGTACTTTAGGCTGTG |
| Mut383 | Mut383-F | TGAATCACTAGCTCAGATTTTTCGGCTATAAGCAAACAACTCCCAGCTGAG |
|  | Mut383-R | CTCAGCTGGGAGTTGTTTGCTTATAGCCGAAAAATCTGAGCTAGTGATTCA |
| 18SRNA | 18SRNA-F | GTAACCCGTTGAACCCCATT |
|  | 18SRNA-R | CCATCCAATCGGTAGTAGCG |
