# Supplementary material for: MiR-224 Targets the 3′UTR of Type 1 5′-Iodothyronine Deiodinase Possibly Contributing to Tissue Hypothyroidism in Renal Cancer
Source: PLoS One. 2011 Sep 2;6(9):e24541. doi: 10.1371/journal.pone.0024541 (PMC3166326; doi:10.1371/journal.pone.0024541)
Supplement: Table S2 — Primers used in analysis of miRNA. RT: primers used in reverse transcription. PCR: primers used in SQ-PCR. F: forward, R: reverse. Uni-amp sequences are bolded. (DOC) [file pone.0024541.s002.doc]

**Table S**2.

| Target miRNA | Primer name | Primer sequence |
| --- | --- | --- |
| hsa-miR-224 | miR224-RT-F | CTTGCATCACCAGAGAACG**AACGGAACC** |
|  | miR224-PCR-F | **GCGAGGT**CAAGTCACTAGTGGT |
| hsa-miR-383 | miR383-RT-F | CTTGCATCACCAGAGAACG**AGCCACAATC** |
|  | miR383-PCR-F | **GCGAGGT**AGATCAGAAGGTG |
| hsa-miR-610 | miR610-RT-F | CTTGCATCACCAGAGAACG**CCAGCACACT** |
|  | miR610-PCR-F | **GCGAGGT**TGAGCTAAATGTGTG |
| hsa-miR-637 | miR637-RT-F | CTTGCATCACCAGAGAACG**TCGCAGAGCCCG** |
|  | miR637-PCR-F | **GCGAGGT**ACTGGGGGCTTTCGGGCT |
| hsa-miR-659 | miR659-RT-F | CTTGCATCACCAGAGAACG**GGGACCCTC** |
|  | miR659-PCR-F | **GCGAGGT**CTTGGTTCAGGGAGG |
| hsa-miR-1202 | miR1202-RT-F | CTTGCATCACCAGAGAACG**CCCACTGCA** |
|  | miR1202-PCR-F | **GCGAGGT**GTGCCAGCTGCAGTG |
| hsa-miR-1266 | miR1266-RT-F | GCATCACCAGAGAACGC**AGCCCTGTTCTA** |
|  | miR1266-PCR-F | **GCGAGGT**CCTCAGGGCTGTAGAACA |
| U6 | U6-F | GCATCACCAGAGAACGAACGCTTCACGAATTTGC |
| reverse primer for analysis of all miRNAs | HindIII-Uni-amp-PCR | CGAGAAGCTTGCATCACCAGAGAACG |
