# Supplementary figures and images for: MiR-224 Targets the 3′UTR of Type 1 5′-Iodothyronine Deiodinase Possibly Contributing to Tissue Hypothyroidism in Renal Cancer
Source: PLoS One. 2011 Sep 2;6(9):e24541. doi: 10.1371/journal.pone.0024541 (PMC3166326; doi:10.1371/journal.pone.0024541)

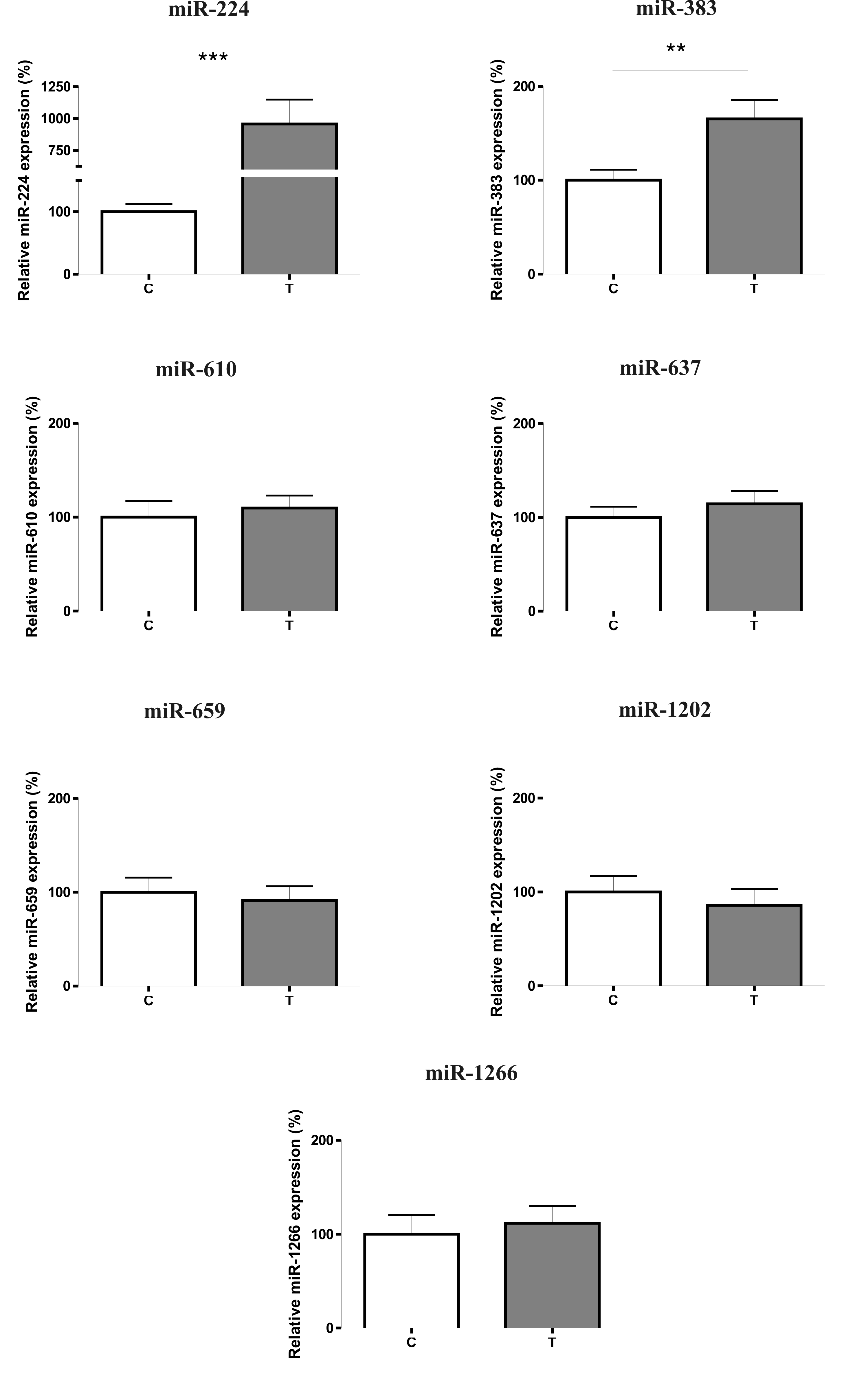

Supplement: Figure S1 — Expression of microRNAs predicted to bind to DIO1 3′UTR in ccRCC. Expression of miR-224, miR-383, miR-610, miR-637, miR-659, miR-1202 and miR-1266 was analyzed in 32 matched pairs of tumor (T) and control (C) samples. SQ-PCR reactions were performed in triplicates. The expression is shown as percentage of control C. Data are given as mean ± SEM (n = 32 for T, n = 32 for C). Statistical analysis was performed using t-test to compare C and T samples. (TIF) [file pone.0024541.s003.tif]

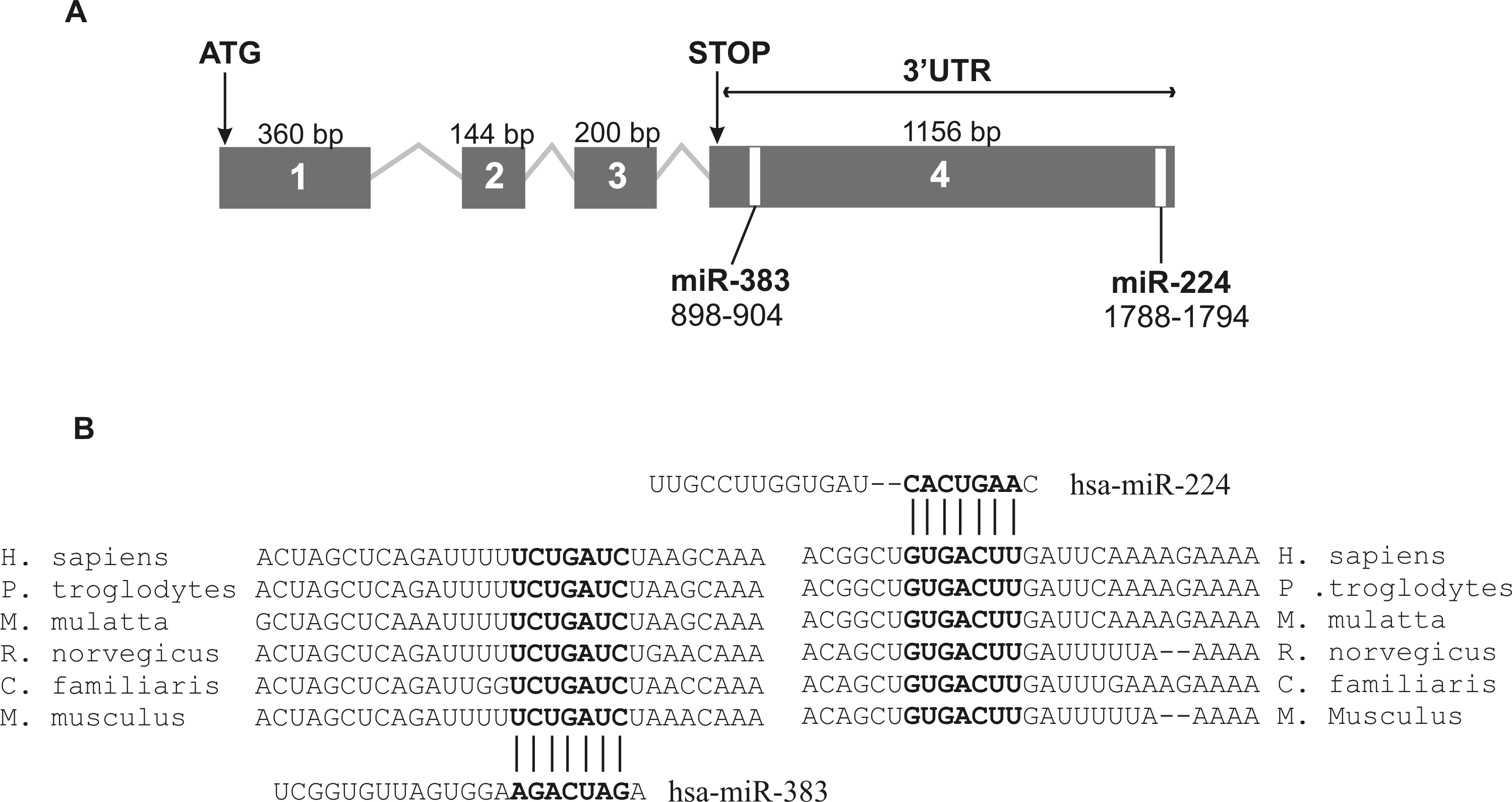

Supplement: Figure S2 — The microRNA target sites in DIO1 3′UTR. A). The structure of the human DIO1 transcript (GeneBank Acc. No. NM_00792.5). The four exons are boxed, numbers above indicate exon length. Stop codon indicates the beginning of 3′UTR. Binding sites of miR-383 and miR-224 are indicated and their positions (miR-383: nt 898-904, miR-224: nt 1788–1794) are given according to DIO1 mRNA sequence (NM_00792.5). B). Bioinformatic prediction of miRNA binding sites in DIO1 3′UTR, conserved among six mammalian species performed with TargetScan5.1. (TIF) [file pone.0024541.s004.tif]
